# Interaction of the endocrine system with inflammation: a function of energy and volume regulation

**DOI:** 10.1186/ar4484

**Published:** 2014-02-13

**Authors:** Rainer H Straub

**Affiliations:** 1 Laboratory of Experimental Rheumatology and Neuroendocrine Immunology, Division of Rheumatology, Department of Internal Medicine, University Hospital, F.J. Strauss-Allee 11, 93042 Regensburg, Germany

## Abstract

During acute systemic infectious disease, precisely regulated release of energy-rich substrates (glucose, free fatty acids, and amino acids) and auxiliary elements such as calcium/phosphorus from storage sites (fat tissue, muscle, liver, and bone) are highly important because these factors are needed by an energy-consuming immune system in a situation with little or no food/water intake (sickness behavior). This positively selected program for short-lived infectious diseases is similarly applied during chronic inflammatory diseases. This review presents the interaction of hormones and inflammation by focusing on energy storage/expenditure and volume regulation. Energy storage hormones are represented by insulin (glucose/lipid storage and growth-related processes), insulin-like growth factor-1 (IGF-1) (muscle and bone growth), androgens (muscle and bone growth), vitamin D (bone growth), and osteocalcin (bone growth, support of insulin, and testosterone). Energy expenditure hormones are represented by cortisol (breakdown of liver glycogen/adipose tissue triglycerides/muscle protein, and gluconeogenesis; water retention), noradrenaline/adrenaline (breakdown of liver glycogen/adipose tissue triglycerides, and gluconeogenesis; water retention), growth hormone (glucogenic, lipolytic; has also growth-related aspects; water retention), thyroid gland hormones (increase metabolic effects of adrenaline/noradrenaline), and angiotensin II (induce insulin resistance and retain water). In chronic inflammatory diseases, a preponderance of energy expenditure pathways is switched on, leading to typical hormonal changes such as insulin/IGF-1 resistance, hypoandrogenemia, hypovitaminosis D, mild hypercortisolemia, and increased activity of the sympathetic nervous system and the renin-angiotensin-aldosterone system. Though necessary during acute inflammation in the context of systemic infection or trauma, these long-standing changes contribute to increased mortality in chronic inflammatory diseases.

## Introduction

Two questions are asked with respect to the ‘interaction of the endocrine system with inflammation’: (a) How does inflammation influence the endocrine system, and does it influence disease? (b) How do hormones influence inflammation and immune cells? Both questions have been extensively addressed over the last decades (for example, [1-3]). Most often, the two questions were posed independently of each other. A theory to integrate both questions has recently been demonstrated in the context of chronic inflammation considering rheumatic diseases.

**Figure 1:**
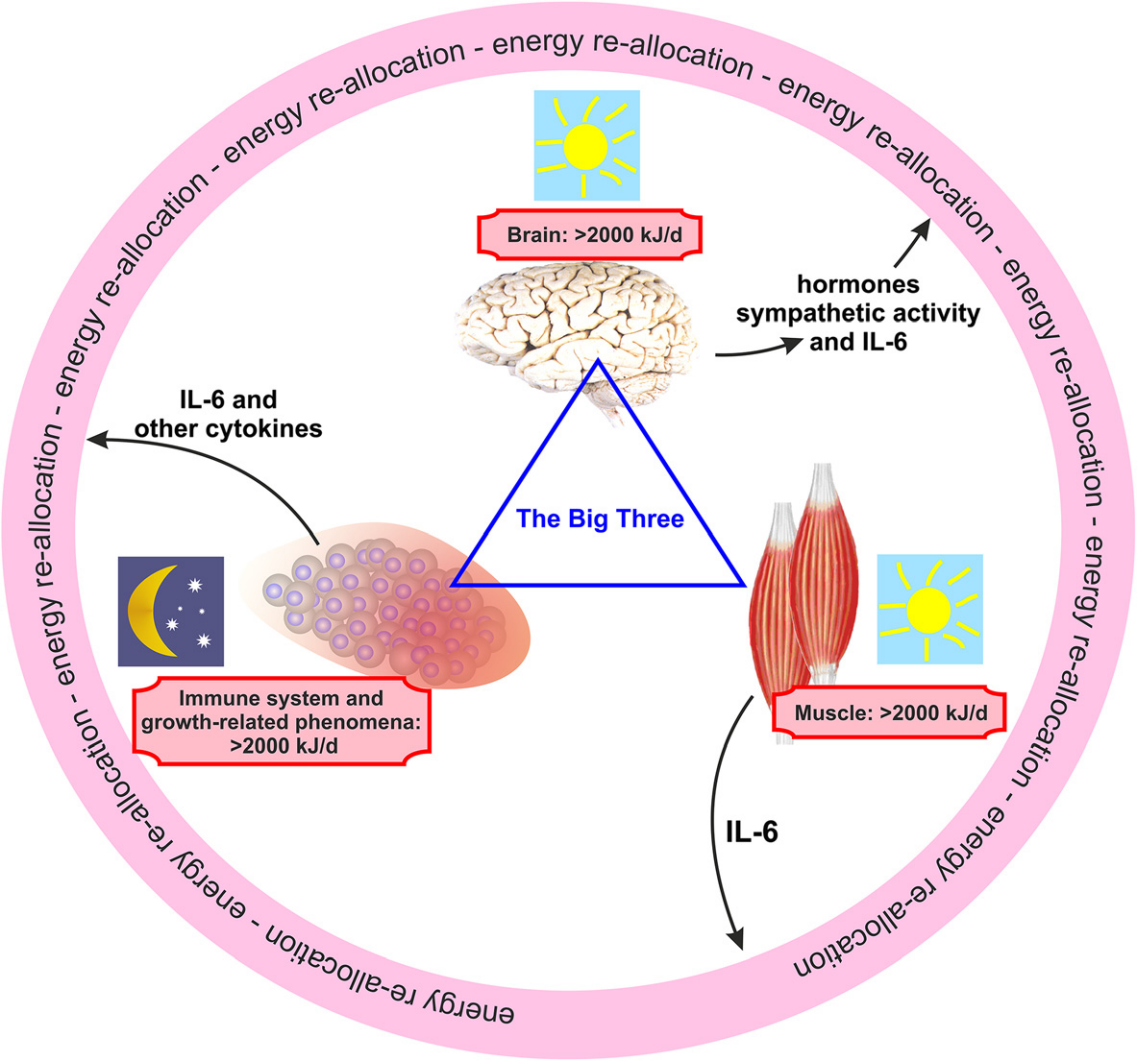
**The three big energy consumers in the body use approximately 2,000 kJ/day under resting conditions.** Calculation of energy expenditure for the widespread immune system is based on a recent publication that mentions 1,600 kJ/day [4]. Growth-related phenomena in adults are added to this number with 400 kJ/day. A demand reaction for energy-rich fuels (pink circular ring) can be started by one of ‘the big three’ mainly using cytokines and hormones, one of which is interleukin-6 (IL-6). The immune system is activated by external triggers such as infectious agents or self-antigens in misguided autoimmunity and thus is independent of the two other big consumers in starting the demand reaction. The brain is activated by external triggers (for example, stressful life events) or by misguided brain function (for example, major depression), and the brain is independent in starting the re-allocation program. An activated muscle demands energy-rich fuels by releasing muscular factors such as IL-6. The muscle is dependent on brain function to start the energy demand reaction. Whereas immune system activation and growth-related processes happen mainly at night, brain function and muscular function are increased during the day (indicated by the moon and the sun symbols).

**Figure 2:**
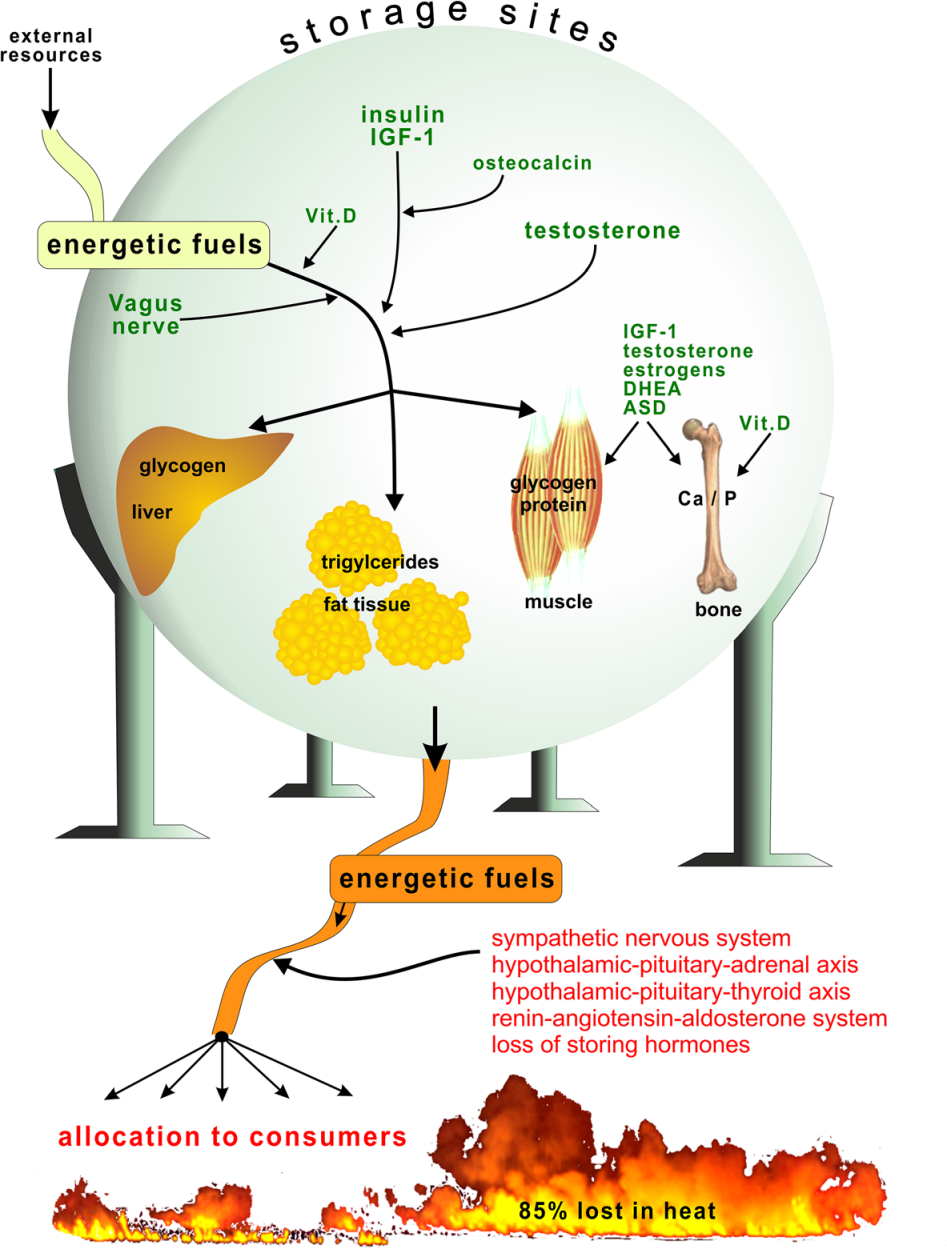
**Storage and release of energy-rich fuels.** Green factors are responsible for storage of energy-rich fuels given in the green bowl. Red factors are relevant for release of energy-rich fuels and allocation to consumers. Storage organs are given (liver: 2,500 kJ as glycogen; muscle: 50,000 kJ as degradable protein; fat tissue as triglycerides: 500,000 kJ; values for an 85-kg person). ASD, androstenedione; Ca, calcium; DHEA, dehydroepiandrosterone; IGF-1, insulin-like growth factor-1; P, phosphorus; Vit. D, vitamin D.

This theory explains neuroendocrine changes in chronic inflammatory diseases (CIDs) on the basis of three pillars: (i) energy-rich fuel allocation is important for an activated immune system [4,5], (ii) increased activity of the water retention system accompanies energy allocation to the immune system [6], and (iii) evolutionary medicine explains that these inflammation-driven energy expenditure programs were positively selected for acute but not chronic systemic inflammation, and chronic use of these programs is highly unfavorable [7]. The platform of the theory is based on the fact that brain, muscle, and immune system use similar amounts of energy-rich fuels (Figure 1). This circumstance necessitates precise regulation of energy-rich fuel allocation to these three systems (Figure 2). The theory says that the activated immune system is the independent stimulus of the observed endocrine and neuronal changes in inflammation as part of an energy re-allocation program.

Since aging, chronic psychological stress, and mental illnesses are also accompanied by chronic smoldering inflammation (such as in CID), many aspects apply to age- and stress-related diseases and mental illness. Chronic smoldering inflammation in humans is already established with elevations of serum IL-6 from normal levels of 1 pg/mL to approximately 10 to 100 pg/mL of serum IL-6, leading to an increase of resting metabolic rate in healthy volunteers [8]. The observable increase of inflammatory cytokines at relatively low serum levels can induce a re-allocation program of energy-rich fuels directed toward the activated immune system. This is confirmed by studies that describe a close interrelation of slightly elevated serum levels of C-reactive protein (IL-6-dependent) and a key element of the energy re-allocation program, namely insulin resistance [9,10].

The review starts with the description of energy storage and energy expenditure hormones. Then, this review briefly summarizes major effects of these hormones on the immune system/inflammation. Finally, it demonstrates changes of these hormonal systems observed in CIDs and consequences thereof. This review deals with energy regulation on a systemic level but not with the question of cellular bioenergetics and ATP generation, which was addressed in a recent publication in the context of rheumatic diseases [5].

## Energy storage hormones

Under normal conditions without activated brain, muscle, or immune system and with undisturbed food intake, energy-rich fuels are stored in specialized organs (adipose tissue, muscle, liver; approximately 560,000 kJ in an 86-kg person; we need 10,000 kJ/day in our sedentary way of life [6]). Under resting conditions and high food supply, energy storage usually outweighs energy expenditure. Under natural conditions in the wild, the situation is balanced in that storage and expenditure are similar (for example, [11]). The following neuroendocrine factors are important for storage.

### Insulin and insulin-like growth factor-1

Insulin is the major hormone responsible for uptake of energy-rich substrates into liver, muscle, and adipose tissue when there is no insulin resistance. It is stimulated mainly by circulating glucose [12]. However, insulin is also directly responsible for growth-related processes [13]. Thus, it is often used in cell culture media, indicating its growth-promoting capacities. This is relevant for leukocytes that, importantly, cannot become insulin-resistant but upregulate GLUT1, GLUT3, and GLUT4 transporters upon activation [14].

Insulin-like growth factor-1 (IGF-1) is an important promotor of muscle growth [15]. Since important glucogenic amino acids like alanine, glutamine, glutamic acid, aspartic acid, and asparagine are deposited in muscular proteins, muscles are important stores of energy-rich fuels that can serve gluconeogenesis [16]. IGF-1 also stimulates growth of adipose tissue and bone [17,18]. Thus, IGF-1 is an important storage hormone for energy-rich fuels and for factors auxiliary to the immune system such as calcium and phosphorus.

### Androgens

Early after the discovery of androgens in the 1930s, the growth-promoting effect of androgens had already been recognized and their anabolic effects on muscle, bone, and hair had been described [19,20]. During World War II, androgens were given to victims of starvation to help restore a positive nitrogen balance typical for anabolic substances. In the 1950 and 1960s, androgens started to be used as doping because of its anabolic effects. The loss of androgens during aging was linked to muscle and bone loss [21]. In addition, testosterone increases insulin sensitivity, and androgen deficiency is linked to the development of type 2 diabetes mellitus [22]. This short summary clearly identifies androgens as anabolic hormones leading to storage of energy-rich substrates and calcium and phosphorus.

### Estrogens

Similar to androgens, estrogens were described to promote bone growth [23], and part of the androgen effect on bone is mediated by aromatization of androgens to estrogens [24]. Thus, estrogens are important in storing calcium and phosphorus in bone. Estrogens influence body fat patterning by inducing the gynoid subcutaneous fat accumulation and by inhibiting the android visceral fat accumulation [25]. However, it seems that estrogens at physiological concentrations inhibit lipogenesis and adiposity as indicated by post-menopausal increase of visceral adipose tissue mass [26]. Estrogens induce insulin sensitivity in the physiological range but can lead to insulin resistance at low (post-menopausal) and high (pregnancy) concentrations [26]. Estrogens also stimulate synthesis and release of pancreatic insulin [26]. The support of insulin is directed mainly toward the muscular compartment because estrogens increase insulin-mediated muscle glucose uptake [26].

In conclusion, the role of estrogens is bi-modal in that both storage and release of energy-rich fuels have been described. This may depend on the balance of estrogen receptor (ER)-type alpha versus ERβ with opposite functions [26]. It can also depend on local estrogen levels and intracellular conversion from androgen precursors, mechanisms described to be relevant in inflammation [27].

### Vitamin D: the D hormone

Since the 1930s, it has been known that vitamin D activates calcium import from the intestinal lumen into circulation, thereby increasing calcium serum levels [28,29]. The second major function of vitamin D is renal re-absorption of calcium and phosphorus [29]. These two functions of vitamin D increase calcium and phosphorus in the circulation. For a long time, the presence of high calcium and phosphorus in circulation was thought to be solely responsible for increased bone mineralization. Today, we know that bone remodeling is also a direct function of vitamin D [29]. Thus, vitamin D is a major hormone for storage of calcium and phosphorus in bone. In addition, vitamin D increases muscle contractile proteins such as actin and troponin C [30]. Polymorphisms in the vitamin D receptor were linked to changes in muscle mass and strength, and vitamin D treatment improves myopathy [30]. This indicates a positive effect on storage of energy-rich fuels in muscles. In summary, vitamin D is responsible for storage of calcium/phosphorus in bone and amino acids in muscle.

### Osteocalcin

Osteocalcin is an osteoblast hormone that can bind calcium ions, regulates bone mineralization and bone turnover, and is used as a biomarker for bone formation [31]. Thus, it is an important hormone for storage of calcium and phosphorus in bone.

In the last decade, different approaches demonstrated osteocalcin as a link between the bone and adipose tissue [31]. Mice lacking osteocalcin displayed decreased pancreatic beta-cell proliferation, glucose intolerance, and insulin resistance [32]. Osteocalcin stimulates insulin expression in pancreatic beta-cells *in vitro* and glucose tolerance *in vivo*[32].

Serum levels of the uncarboxylated form of osteocalcin are associated with improved glucose tolerance and enhanced pancreatic beta-cell function in middle-aged men [33]. Serum osteocalcin was negatively correlated with fasting insulin and ‘homeostasis model assessment of insulin resistance’ [34]. Several other reports link high osteocalcin levels to insulin sensitivity [31], but studies in humans have not yet shown direct causal effects [35]. Nevertheless, these studies indicate that osteocalcin supports insulin function and thus storage of energy-rich fuels in adipose tissue and muscle.

Mouse models with loss- or gain-of-function mutations in the osteocalcin gene (*BGLAP*) suggested that osteocalcin is responsible for the regulation of fertility in males only [36]. Leydig cells treated with uncarboxylated osteocalcin showed increased testosterone synthesis [36]. This might demonstrate positive cross-talk of two storage hormones in the energy storage network (Figure 2).

Finally, one of the major hormones to release energy-rich fuels from storage sites, namely glucocorticoid, reduces osteocalcin levels in that it inhibits osteoblast function [37]. This is another indication of the storage function of this hormone that can be switched off by a hormone of the energy expenditure network.

### Vagus nerve

In the fasting situation, vagal afferents are important in transferring hepatoportal information on low blood glucose and low levels of other nutrients to the dorsal vagal complex, leading to hunger signals, inhibition of sympathetic activation, inhibition of efferent vagal activation, hypometabolism, and hypothermia [38-40]. In acute systemic infection without food intake, this behavior protects energy stores.

In the feeding situation, vagal afferents together with gastrointestinal hormones such as cholecystokinin transmit signals to the dorsal vagal complex, leading to satiety signals, activation of the sympathetic nervous system (SNS) (short-lived post-prandial thermogenesis and hypermetabolism), activation of efferent vagal nerve fibers (propulsive motility and secretion of exocrine and endocrine pancreatic and gastrointestinal factors), hyperinsulinemia, and thus storage of energy-rich fuels [38-41]. Although the SNS post-prandially induces short-lived hypermetabolism, the net effect of the vagus nerve leads to energy-storage largely because of hyperinsulinemia. This is supported by an important experiment in the early 1990s. Lesioning of the SNS headquarters in the hypothalamus, the ventromedial hypothalamic nucleus, leads to hypoactivity of the SNS and hyperactivity of the efferent vagus nerve with hyperinsulinemia and obesity [42]. Thus, in the fasting and in the feeding situation, the vagus nerve is responsible for energy storage.

## Energy expenditure hormones

Energy expenditure hormones are typically released upon stressful events such as hypoglycemia or other forms of stress such as acute inflammation or trauma. This can happen in acute inflammatory situations such as systemic infectious diseases, which induce an energy re-allocation program with three major pathways: (i) release of energy-rich fuels from storage sites into circulation (liver glucose, muscle amino acids, glycerol, and free fatty acids from fat tissue), (ii) inhibition of uptake of energy-rich fuels into liver, muscle, and fat tissue by intentionally induced insulin resistance, and (iii) inhibition of growth-related and reproductive functions [4]. Similar programs are used in CIDs.

### Cortisol

Cortisol was demonstrated to be a muscle-catabolic factor inducing rapid release of amino acids from muscle [43]. Glucocorticoids stimulate overall lipolysis at the whole-body level [44], but glucocorticoids may specifically inhibit abdominal lipolysis because chronic hypercortisolemia secondary to Cushing’s syndrome is characterized by distinct abdominal obesity. Recent data point toward cortisol-induced increase of visceral fat on the basis of visceral 11β-hydroxysteroid dehydrogenase type 1 availability [45]. Thus, cortisol would support lipolysis and re-allocation of lipids to visceral stores and elsewhere.

Cortisol is an important stimulator of glycogenolysis and gluconeogenesis in the liver [46]. Cortisol inhibits bone formation by blocking osteoblasts, decreasing osteocalcin levels, and interfering with several other pathways [37]. Cortisol supports insulin resistance so that energy-rich substrates cannot be taken up into muscle, liver, and fat tissue [47].

In conclusion, cortisol via many independent pathways belongs to the network of energy-expenditure hormones, an increase of which leads to rapid allocation of energy-rich fuels to the immune system. However, a program with long-term elevation of cortisol is not positively selected, because of the danger of immunosuppression and sepsis.

### Noradrenaline/adrenaline-sympathetic nervous system

The SNS innervates the liver and supports hepatic glycogenolysis and gluconeogenesis, leading to release of glucose [48]. With respect to another important energy-rich fuel, namely free fatty acids, the SNS induces lipolysis in brown and white adipose tissue [49]. The SNS is also responsible for release of the third group of energy-rich fuels, namely amino acids. Neurotransmitters of the SNS can induce muscle breakdown which is β-adrenoceptor-mediated [50]. It was suggested that the SNS is a regulator of muscle catabolism [51]. The SNS inhibits insulin secretion leading to little glucose provision to muscles, liver, and fat tissue [52]. Furthermore, the SNS drives insulin resistance in target organs such as muscle, liver, and fat tissue so that uptake of energy-rich fuels is inhibited [53]. The SNS activates glucagon secretion from the pancreas [54], thereby helping to provide glucose to the activated immune system. In addition, the SNS is a key element of bone turnover leading to net bone loss [32]. All these functions of the SNS are related to allocation of energy-rich fuels and calcium and phosphate to an activated immune system.

Since cortisol and noradrenaline are often cooperative in their individual functions by increasing the respective signaling pathway of the β-adrenoceptor and glucocorticoid receptor type α (for example, [55,56]), these two hormones support each other in release of energy-rich fuels or calcium/phosphorus from stores. Furthermore, the SNS is the important stimulator of the renin-angiotensin-aldosterone system (RAAS) leading to water and sodium retention (see below).

### Growth hormone

In adults, the growth-promoting effects of growth hormone are mediated mainly via IGF-1 [57]. Growth hormone via IGF-1 increases net whole-body protein synthesis and, thus, growth hormone has some anabolic effects [57], which might change during inflammatory illness because of growth hormone receptor resistance and loss of pulsatile growth hormone secretion [58-61].

Concerning effects of IGF-1 and growth hormone on metabolism, IGF-1 often demonstrates opposite effects to growth hormone [57]. IGF-1 increases insulin sensitivity, decreases hepatic glucose production, and stimulates muscle glucose uptake, whereas growth hormone increases insulin resistance, increases hepatic glucose production, and reduces muscular and fat tissue glucose uptake [57]. Consequently, growth hormone increases blood glucose and, thus, this hormone is one of the major counter-regulatory hormones to insulin.

While IGF-1 has no influence on lipolysis in adipose tissue, growth hormone induces lipolysis and reduces lipogenesis [57]. Thus, growth hormone in contrast to IGF-1 has many catabolic effects leading to provision of energy-rich substrates to an activated immune system in inflammation. In addition, growth hormone has anti-natriuretic effects, thereby adding to water and sodium retention [62].

### Thyroid gland hormones

Thyroid hormone levels in the normal range are inversely correlated with body weight in women and men, indicating catabolic effects [63]. Thyroid hormones accelerate metabolism, increase lipolysis, stimulate hepatic gluconeogenesis, induce thermogenesis, increase the Cori cycle (the cycle of glucose-lactate-glucose between liver and glucose-demanding tissue), decrease glycogen stores, accelerate insulin degradation, and increase GLUT4 glucose transporters in the skeletal muscle and monocytes [64-66].

Various functions of thyroid hormones on metabolism depend on concomitant signaling of thyroid hormones and catecholamines [67]. Cooperativity of the two hormone pathways depends on signaling through cyclic AMP response elements and thyroid hormone response elements on the promoter of many genes (for example, phosphoenolpyruvate carboxykinase, the key enzyme of gluconeogenesis) [67]. Catecholamines increase the set point for feedback inhibition of the hypothalamic-pituitary-thyroid axis by the biologically active tri-iodothyronine (T3), leading to increased thermogenesis [65]. Thus, thyroid hormones are major energy expenditure hormones.

A euthyroid status is important for normal bone development during growth and for maintenance of bone in adulthood [68]. Population studies indicate that hormone deficiency and hormone excess are associated with increased bone loss and fracture risk [68]. Elevated T3 induces catabolic effects on bone as substantiated in hyperthyroidism [68]. Thus, T3 at higher levels leads to provision of calcium and phosphorus.

While thyroid hormones stimulate the RAAS and sodium retention, they also directly increase heart rate, cardiac output, blood pressure, water intake, and various ion channels and transporters, leading to increased glomerular filtration [69]. Patients with hyperthyroidism often show polyuria but this is probably related to increased water intake [69]. However, if one studies glomerular filtration rate in relation to kidney weight (thyroid hormones increase kidney weight), then thyroid hormones decrease glomerular filtration and increase water and sodium retention [70].

### The renin-angiotensin-aldosterone system

Apart from its major function of sodium and water retention, the RAAS was found to induce insulin resistance on local and systemic levels, which explains why therapies that interfere with the RAAS reduce the incidence of type 2 diabetes mellitus [71-73]. Angiotensin II stimulates glycogenolysis and gluconeogenesis [74]. It seems that hormones of the RAAS exert these effects via intracellular induction of reactive oxygen species [71,72]. Angiotensin II via the angiotensin receptor type 1 was directly involved in insulin receptor inhibition [73].

In addition, angiotensin II stimulates osteoclasts *in vitro* and *in vivo*[75,76], and treatment of hypertensive patients with angiotensin-converting enzyme (ACE) inhibitors was related to a lower risk of bone fractures and a higher bone mineral density [77,78]. It is highly interesting that vitamin D, which is often low in CIDs, is a negative regulator of the RAAS, so that low levels of vitamin D would support insulin resistance together with bone loss [79].

Angiotensin II stimulates noradrenalin release from sympathetic nerve terminals, which explains why some effects of the RAAS on systemic energy regulation and bone metabolism are similar to effects of the SNS [73]. Thus, similar to the SNS with its major neurotransmitters noradrenaline/adrenaline, angiotensin II, a major hormone of the RAAS, supports re-allocation of energy-rich fuels and calcium/phosphorus to an activated immune system.

### Summary 1

Table 1 summarizes the effects of different types of hormones on energy storage and energy expenditure. It turns out that there are two major networks, one that stores energy-rich fuels and one that releases energy-rich fuels upon stressful life events such as acute inflammation (systemic infectious disease or injury), psychological stress, trauma/hemorrhage, pain, and mental illness. The condition of chronic smoldering inflammation during aging or in obesity most probably influences the two networks in a similar but more protracted way over decades. The chronic misuse of the energy expenditure system leads to the known systemic disease sequelae in chronic inflammatory diseases (see below).

**Table 1:** Summary of energy storage and energy expenditure hormones

	**Fat tissue**	**Muscle**	**Liver**	**Bone**	**Kidneys**
**Energy storage hormones**					
Insulin	Uptake^a^	Uptake^a^	Uptake^a^		
Insulin-like growth factor-1^b^	Growth	Growth	Uptake^a^	Growth	
Androgens^b^		Growth		Growth	
Estrogens^b^	Gynoid fat distribution	Glucose uptake^a^		Growth	
Vitamin D		Growth		Growth	Calcium/phosphorus retention
Osteocalcin^b,c^		Glucose uptake^a^	Glucose uptake^a^	Growth	
Vagus nerve	Uptake^a^		Uptake^a^		
**Energy expenditure hormones**					
Cortisol^d^	Release^a^	Release^a^	Release^a^	Release^a^	Water/sodium retention
Sympathetic nervous system (noradrenaline/adrenaline)^d^	Release^a^	Release^a^	Release^a^	Release^a^	Water/sodium retention
Growth hormone^d^	Release^a^	Release^a^	Release^a^	Growth via IGF-1	Water/sodium retention
Thyroid hormones (T3)^d^	Release^a^	Release^a^	Release^a^	Release^a^	Water/sodium retention
RAAS^d^			Release^a^	Release^a^	Water/sodium retention

^a^Uptake/release of energy-rich fuels into/from respective tissue; ^b^increase of insulin sensitivity; ^c^support of androgens; ^d^decrease of insulin sensitivity (induction of insulin resistance). IGF-1, insulin-like growth factor-1; RAAS, renin-angiotensin-aldosterone system; T3, tri-iodothyronine.

## Hormone effects on inflammation and observed changes in chronic inflammation: consequences for energy and volume regulation

### Insulin and insulin-like growth factor-1

In earlier years, the direct effect of insulin on immune cells was demonstrated to be pro-inflammatory, mainly by supporting proliferation of immune and other cells [80]. In recent years, insulin was categorized as an anti-inflammatory hormone because it is able to remove energy-rich fuels such as glucose and free fatty acids from circulation that would nourish the immune system [81,82]. Although this latter concept seems reasonable with intact insulin signaling, it will not work under insulin resistance of liver, muscle, and fat tissue. Thus, the balance between systemic anti-inflammatory insulin effects and local pro-inflammatory insulin effects will influence the role of this hormone in a given situation. It is proposed that insulin resistance is the critical determinant of the pro- or anti-inflammatory effect of insulin, because only liver, muscle, and fat tissue, but not leukocytes, become insulin-resistant [14].

IGF-1 was demonstrated to have mainly pro-inflammatory effects [83,84]. The aspects of IGF-1 are stimulation of hematopoiesis, T and B lymphopoiesis, increase of natural killer cell activity, priming of macrophages and neutrophils for radical production, increase of TNF production from macrophages, sensitization for mitogen stimulation, and enhanced primary antibody responses *in vivo*[83].

**Figure 3:**
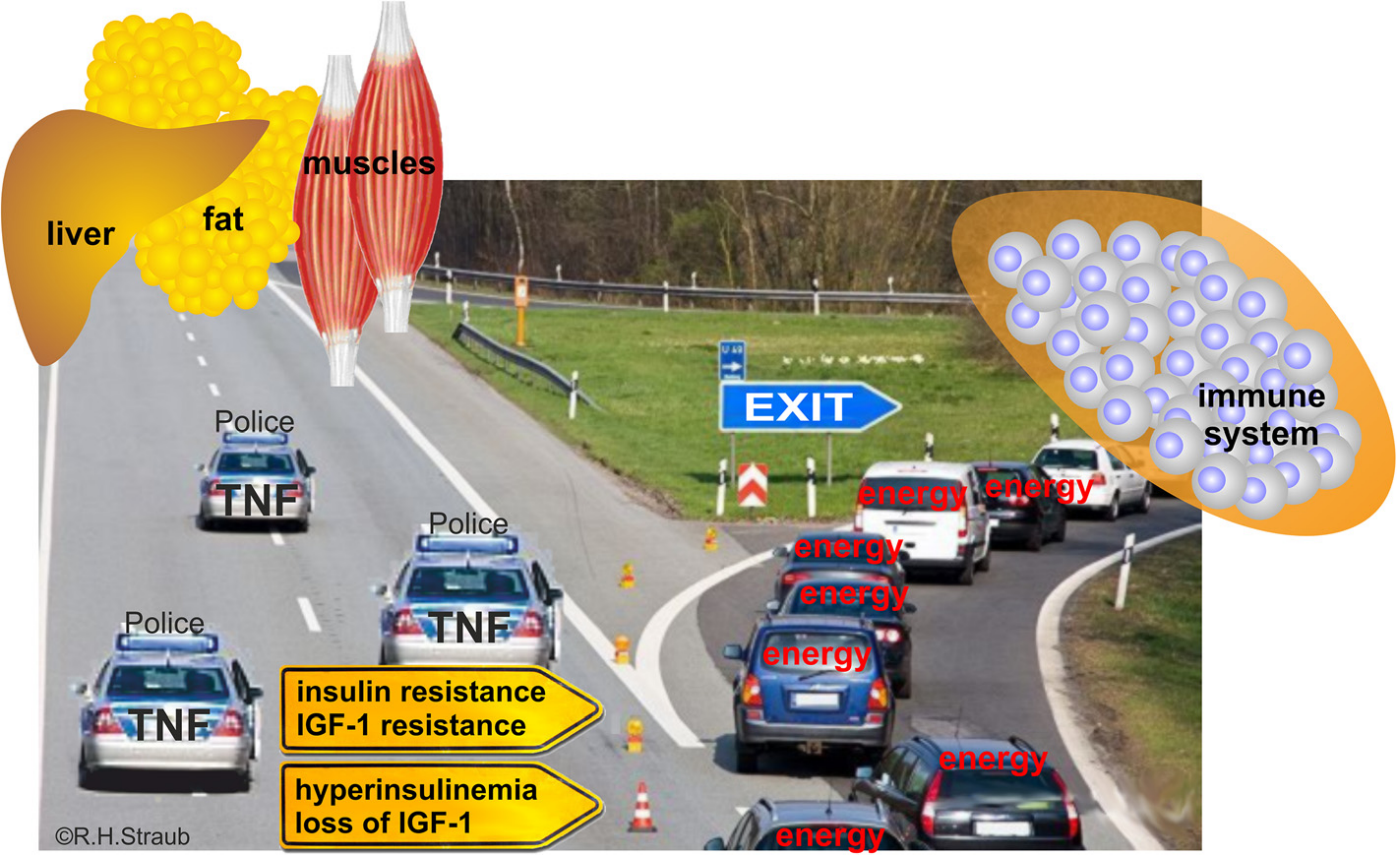
**Schematic representation of the consequences of insulin and insulin-like growth factor-1 (IGF-1) signaling alterations.** Pro-inflammatory factors such as tumor necrosis factor (TNF) reduce signaling of insulin and IGF-1 and production of IGF-1 from liver (for example, [91]). This program affects liver, adipose tissue, and muscle, but not immune cells, because they cannot become insulin-resistant. The consequence is a deviation of energy-rich fuels from storage sites (liver, adipose tissue, and muscle) to the activated immune system and inflammatory tissue.

In CIDs such as rheumatoid arthritis (RA) and systemic lupus erythematosus (SLE), hyperinsulinemia and insulin resistance were described [85-87]. In addition, IGF-1 resistance was described in patients with RA [60], and IGF-1 levels are typically decreased in chronic inflammation [88-90]. Thus, both pathways through insulin and IGF-1 receptors are not intact in CIDs. For insulin, uptake of energy-rich fuels into liver, muscle, and fat will be diminished, but direct activation of local immune cells will still be possible (Figure 3). Loss of IGF-1 will be associated with a cachectic situation due to the growth-promoting activity of this hormone in muscle. Thus, loss of IGF-1 and IGF-1 resistance will diminish the stores for energy-rich fuels and auxiliary factors in muscles, fat tissue, and bone, thereby serving the activated immune system (Figure 3).

### Androgens and estrogens

Whereas androgens are mainly anti-inflammatory [92,93], estrogens have a bi-modal pro- and anti-inflammatory role. Estrogen effects depend on several recently summarized criteria [27]: (a) the immune stimulus (foreign antigens or autoantigens) and subsequent antigen-specific immune responses (for example, T cell inhibited by estrogens versus B cell activated by estrogens), (b) the cell types involved during different phases of the disease, (c) the target organ with its specific microenvironment, (d) timing of 17β-estradiol administration in relation to the disease course (and the reproductive status of a woman), (e) the concentration of estrogens, (f) the variability in expression of ERα and ERβ depending on the microenvironment and the cell type, and (g) intracellular metabolism of estrogens leading to important biologically active metabolites with quite different anti- and pro-inflammatory function. Thus, B cell-dominated immune reactions that are supported by T helper type 2 immune responses are stimulated, whereas T helper type 1 and macrophage-dominated immune responses are inhibited.

In CIDs of different etiology, serum androgens are very low [94-96], but estrogen levels remain relatively normal as a consequence of increased conversion of androgens into estrogens in inflamed tissue [97]. Androgen loss is particularly evident for the adrenal androgen dehydroepiandrosterone sulfate, the major precursor of androgens in post-menopausal women and older men. Loss of androgens together with a loss of and resistance to IGF-1 is a very critical factor for cachexia and bone loss, leading to a loss of energy storage sites in skeletal muscle and bone.

Since estrogens can be produced locally [97] and since estrogens support pre-adipocyte proliferation and local fat tissue growth [25,98] as well as lipoprotein lipase necessary to store lipids at low estrogen concentrations [99], local estrogen levels might determine regional accumulation of fat tissue such as in synovial fat tissue of patients with RA or juxtaintestinal fat tissue in patients with Crohn disease (called creeping fat). Such a local accumulation would serve regional storage of energy-rich fuels.

### Vitamin D and osteocalcin

Vitamin D was described to foster many aspects of innate immunity but inhibits adaptive immunity toward a T helper type 1 and T helper type 17 direction [100]. Vitamin D is an important factor in the development of tolerogenic dendritic cells and T regulatory lymphocytes [101].

Hypovitaminosis D is a general phenomenon in many CIDs [100]. This might be due to little exposure to sunshine, decreased conversion of endogenous vitamin D precursors, disturbed resorption of vitamin D precursors in the gut, and/or sickness behavior-dependent malnutrition. Thus, loss of vitamin D may support the inflammatory process but also general osteoporosis, which would serve the immune system by providing calcium and phosphorus.

For osteocalcin, no effects on immune responses and inflammatory cells have been described in the literature. In regard to serum levels of osteocalcin, decreased, increased, or normal levels have been described in patients with CIDs [102-106]. Thus, with respect to osteocalcin, no clear picture emerges as to the help of this hormone in the energy regulation of CIDs.

### Vagus nerve

The vagus nerve was reported to have important anti-inflammatory activities in acute inflammation due to inhibition of TNF [107]. This can be a favorable ‘cholinergic reflex’ that might inhibit inflammation in CIDs as recently noticed [107]. However, chronic inflammation such as in RA is accompanied by vagal hypoactivity and sympathetic hyperactivity [108-110]. Thus, an anti-inflammatory and energy-storing function of the vagus nerve is probably not available. Both aspects would support ongoing inflammation in CIDs.

### Cortisol and the sympathetic nervous system

Cortisol is an anti-inflammatory hormone on most occasions [111]. Thus, a long-standing increase of serum cortisol levels after acute stressful events such as infectious disease would be unfavorable because of the danger of sepsis. However, after acute activation of the hypothalamic-pituitary-adrenal (HPA) axis with short-term increase of serum cortisol, there is a rapid reduction of this major anti-inflammatory hormone, best recognized after repeated injections of cytokines [112]. Similarly, in untreated CIDs, serum levels of cortisol are usually normal or somewhat higher [113], but they are inadequately low to suppress the ongoing inflammatory process. Somewhat elevated cortisol levels would serve insulin resistance and glucose/free fatty acid provision to the activated immune system, but at these levels one does not expect many anti-inflammatory effects (inadequate levels in relation to inflammation). Slightly elevated cortisol might also support bone breakdown in order to deliver auxiliary factors such as calcium and phosphorus to activated immune cells.

In contrast to transient cortisol increase, there exists a long-standing activation of the SNS in CIDs [110]. There seems to be a dissociation between high activity of the SNS and relatively normal activity of the HPA axis in CIDs [114,115]. Since sympathetic neurotransmitters can have anti-inflammatory effects on monocytes, macrophages, natural killer cells, neutrophils, and T helper type 1 lymphocytes via β2-adrenergic receptors, increased activity of the SNS might be favorable in local inflammation [116]. However, it was observed that local density of sympathetic nerve fibers is markedly reduced in inflamed tissue of different CIDs and in secondary lymphoid organs of animals with CIDs, which is a pro-inflammatory signal recently summarized [116].

Thus, a higher activity of the SNS supports provision of energy-rich fuels on a systemic level but probably has no favorable anti-inflammatory effects in inflamed tissue. This can also be the reason for a disease-propagating role of the SNS in animal models of arthritis (summarized in [116]). In addition, an elevated sympathetic nervous tone stimulates sodium and water retention and bone loss, which support the activated immune system.

### Growth hormone

Growth hormone accelerates recovery of the immune system following transplantation of various cell types, and it replenished the severely affected T-cell compartment in patients with HIV [117]. Treatment with drugs that stimulate growth hormone secretion or treatment with growth hormone can have positive effects in restoring aspects of the aged immune system [117]. Immune cells carry the growth hormone receptor, and growth hormone signaling involves JAK2-STAT-Ras-MAPK pathways that are shared by cytokine signaling pathways (summarized in [117]).

Growth hormone given to healthy volunteers slightly but significantly increased serum TNF and serum IL-6 [118]. Growth hormone was made responsible for the chronic smoldering inflammation during aging, which can be studied in growth hormone pathway-deficient mice that demonstrate less inflammation and increased longevity [119]. Furthermore, growth hormone primes neutrophils for production of lysosomal enzymes and superoxide anions, supports survival of memory T cells, increases immunoglobulin secretion of B cells, and stimulates thymulin secretion by thymic epithelial cells, natural killer cell activity, phagocytosis, oxidative burst, and killing capacity of neutrophils or macrophages [120]. Transgenic mice overexpressing growth hormone or its receptor exhibit overgrowth of the thymus and spleen and display increases in mitogenic responses to Concanavalin A [120].

Growth hormone serum levels were lower, normal, or slightly elevated in patients with RA and SLE (summarized in [121]). Thus, in patients with CIDs, growth hormone serum levels behave similarly to cortisol serum levels. In other words, there is no clear increase or decrease of hormone levels in serum. One might argue that, because of unchanged serum levels, these hormones are not much involved. However, the anti-inflammatory role of endogenous cortisol was visualized in CIDs by blocking endogenous cortisol production with metyrapone [122]. We learned that growth hormone has important immunostimulating effects, and the question appears whether inhibition of growth hormone release by somatostatin also demonstrates effects of the endogenous hormone in CIDs, similar to endogenous cortisol when blocked with metyrapone.

Open therapies with the growth hormone inhibitor somatostatin in small studies demonstrated anti-inflammatory effects such as reduction of synovial membrane thickness [123] and improved clinical symptoms such as morning stiffness and other American College of Rheumatology criteria in RA [124,125]. Although somatostatin has direct suppressive effects on immune cells and nociceptive nerve fibers [126], somatostatin might also block growth hormone release on a systemic level in the pituitary gland. Thus, one can hypothesize that anti-inflammatory effects of somatostatin or other growth hormone blockers are expected on the basis of inhibition of energy expenditure and inhibition of immunostimulation, two functions of growth hormone. This sounds pretty reasonable, but experimental proof in this complex growth hormone-IGF-1 system is necessary.

During growth in children and adolescents, the situation might be quite different because energy-rich fuels like glucose and amino acids and calcium/phosphorus are stored in muscle and bone (they are not provided to the active immune system). Growth hormone might be judged in a different way in juvenile forms of CIDs because of anabolic effects on muscle and bone growth [127]. Indeed, in juvenile forms of CIDs, growth hormone can have favorable growth-promoting but not anti-inflammatory effects (summarized in [127]). From this point of view, one can hypothesize that growth hormone effects depend on a balance between storage versus expenditure of energy-rich fuels and calcium/phosphorus. Growth hormone might shift this balance toward energy storage in children and energy expenditure in adults.

### Thyroid hormones

Thyroid hormones induce oxygen radical production in neutrophils [128], IFN-γ-stimulated major histocompatibility complex class II expression [128], IL-6, IL-8, and IL-12 secretion from different cell types [128], lymphocyte proliferation [128], IFN-γ-stimulated natural killer cell activity [128], and superoxide anion production in human alveolar neutrophils and macrophages [129]. Thyroid hormones are required for normal B-cell production in the bone marrow [130]. These genomic effects are complemented by non-genomic effects of thyroid hormones [128]. Thyroid hormones can bind to the integrin αvβ3 to switch on a cascade of signaling events through MAPK, phospholipase C, proteinkinase Cα, ERK1/2, and/or hypoxia-inducible factor-1, leading to enhanced cytokine and growth factor action and angiogenesis [128]. Apart from classic actions of thyroxine (T4) and T3, the thyroid gland-stimulating hormone (TSH) has many supportive effects on the immune system [120]. Although thyroid hormones have also some anti-inflammatory actions, usual concentrations of hormones of the hypothalamic-pituitary-thyroid gland axis exert many stimulatory effects on the immune system and inflammation. The question remains whether thyroid hormones like the biologically active T3 are really elevated during acute and chronic inflammation.

Elevation of systemic inflammation such as during injury, inflammation, or starvation leads to the non-thyroidal illness syndrome, which has the following features [131]: (a) downregulation of hypothalamic thyrotropin-releasing hormone; (b) lowered secretion of TSH, free T4, and free T3; (c) decreased levels of circulating free T3 due to decreased peripheral T4→T3 conversion; and (d) increased metabolism of biologically active T3 to inactive reverse T3. All mechanisms lead to inhibition of the hypothalamic-pituitary-thyroid gland axis.

Cytokines play an important role in this sequence as demonstrated for IL-6. After injection of IL-6 into healthy volunteers, T4 and free T4 increased after 4 hours but T3 levels were reduced 24 hours later, which indicates a rapid downregulation of this biologically active hormone by IL-6 [132].

Although not many studies in chronic rheumatic diseases exist, it seems likely that levels of thyroid hormones are low as demonstrated in SLE and Kawasaki disease [133,134]. However, it remains unclear whether this is a direct effect of increased circulating cytokines (as detected in non-thyroidal illness syndrome) or secondary anti-thyroid autoimmunity with functional defects. A recent investigation in patients with RA found a decrease in TSH levels during TNF-neutralizing therapy [135]. This might be interpreted as a consequence of TNF-induced reduction of peripheral thyroid hormones and, consequently, upregulation of TSH, but the exact mechanisms remain enigmatic. In summary, in chronic and acute inflammation, there seems to exist a rapid downregulation of the hypothalamic-pituitary-thyroid gland axis.

Importantly, recent experimental studies have shown that downregulation of the central part of this axis observed during acute and chronic inflammation does not necessarily induce decreased thyroid hormone levels in key metabolic organs such as liver, muscle, and adipose tissue (summarized in [136]). The differential regulation of local thyroid hormone availability depends mainly on expression of activating (D1 or D2) and inactivating (D3) deiodinases [136].

For example, during acute inflammation, in the muscle, the hormone-activating deiodinase D2 increases whereas the hormone-inactivating D3 decreases, which would lead to higher muscular T3 levels, resulting in increased breakdown of energy-rich substrates in the muscle only [136]. This is different in chronic inflammation, where D2 and D3 are elevated and, as was described, the net effect on T3 is a reduction in active T3 [136]. A similar concept exists in the liver, but the exact pathways are far from clear [136]. Local deiodinase expression has not been tested in adipose tissue during inflammation, so thyroid hormone availability is not known in this compartment.

In granulocytes, the inactivating D3 is highly expressed [136]. In these cells, the release of inorganic iodide was related to improved killing of bacteria [136]. This is a very attractive concept because it might well explain the stimulating effects of thyroid hormones on phagocytosis and killing independent of genomic effects via thyroid hormone receptors.

In conclusion, the hypothetical sequence of events during inflammation might be as follows: (a) there is a rapid increase of thyroid hormones for the first 4 hours; (b) then, a rapid downregulation of the hypothalamic-pituitary-thyroid gland axis is established (as observed as low T3 non-thyroidal illness syndrome); (c) this is accompanied by differential expression of deiodinases with relatively normal local T3 in metabolic organs, which might serve the activated immune system by breakdown of energy-rich substrates (known for muscle); and (d) activated granulocytes would be nourished by these circulating substrates and, in parallel, increase D3 expression to provide inorganic iodide necessary to kill bacteria.

### Angiotensin II

While systemic effects of angiotensin II are related to hemodynamic and metabolic functions (see above), local RAAS pathways support pro-inflammatory, proliferative, and profibrotic activities via angiotensin type (AT)1 receptors that couple to G proteins of the type Gq and Gαi (recently summarized in [137]). AT2 receptors also couple to Gαi proteins, a G protein that supports pro-inflammatory pathways. In different organs such as kidney, heart, and vasculature, angiotensin II induces an inflammatory response by fostering the expression of pro-inflammatory chemokines, responsible for tissue accumulation of immunocompetent cells [137]. Angiotensin II via AT1 receptors is also a pro-inflammatory factor in a lupus mouse model [138]. ACE inhibitors of different types reduce severity of collagen type II-induced arthritis [139,140].

Acute infectious disease leads to upregulation of the RAAS system in a mouse model of cytomegalovirus infection [141]. Injection of lipopolysaccharide into rats increased activity of the RAAS system [142]. Patients with sepsis demonstrate increased activity of the RAAS [143]. ACE is upregulated in synovial tissue of patients with RA, leading to higher availability of angiotensin II in inflamed joints [144]. Although the pro-inflammatory role of angiotensin II is well established, very few studies have addressed serum levels of hormones of the RAAS in humans. Two Russian studies identified increased levels of angiotensin II and aldosterone in patients with RA and SLE, but this awaits further confirmation [145,146].

In conclusion, all of these findings indicate that the RAAS is activated in acute and chronic inflammation. Since the RAAS exerts pro-inflammatory effects in addition to its function as an energy expenditure hormonal system, it is perfectly able to support the re-allocation of energy-rich fuels to the activated immune system. In addition, water retention with these hormones will be of outstanding importance, possibly leading to volume overload.

## Conclusions

For some time, the role of energy expenditure hormones and energy storage hormones has been known in critically ill patients with acute inflammation [147]. Transfer of knowledge from acute inflammation to CIDs was blocked by the understanding that quite different pathways might be activated. In addition, most CID researchers worked in the field of aberrant immune activation or autoimmunity, but not many people devoted time to the research field of neuroendocrine immune mechanisms in CIDs. Considerations of evolutionary medicine paved the way to understand that many neuroendocrine pathways used in acute inflammatory illness are similarly used in CIDs [6]. However, the long-term use of these pathways is harmful.

Table 2 identifies effects of individual energy storage and energy expenditure hormones on the immune system and inflammation. This table also summarizes observed changes in CIDs and consequences of long-term application of these adaptive programs, positively selected for short-lived inflammation. It turns out that many neuroendocrine pathways support immune activation (third column in Table 2), which in light of autoimmunity or immunity toward harmless microbes in the gut/skin/respiratory tract is a misused program. In addition to inducing immune activation, many reported neuroendocrine mechanisms induce insulin resistance, cachexia, osteoporosis, and volume overload/hypertension (water retention). In epidemiological studies, these elements were related to higher mortality and morbidity in CIDs. Thus, long-standing use of neuroendocrine pathways is in itself a disease-aggravating etiologic factor.

**Table 2:** Changes of the hormonal systems in chronic inflammatory rheumatic diseases

	**Effect on immune system/inflammation**	**Observed changes in chronic inflammatory rheumatic diseases**	**Long-term consequences**
**Energy storage hormones**			
Insulin	Direct support of immune cells	Hyperinsulinemia, insulin resistance	Insulin resistance, cachexia,
	Pro-inflammatory in a state of systemic insulin resistance		Stimulation of sympathetic nervous system^a^
	Leukocytes do not become insulin-resistant		Immune activation
Insulin-like growth factor-1	Support of innate and adaptive immunity [83,84]	Low IGF-1, IGF-1 resistance	Cachexia, osteoporosis, immune activation
Androgens	Inhibition of immune system and inflammation [148]	Hypoandrogenemia	Cachexia, loss of fertility, osteoporosis
			Insulin resistance, immune activation
Estrogens	Bi-modal role: support of B lymphocytes and T helper type 2; inhibition of macrophages, natural killer cells, and T helper type 1 (see [27])	Normal peripheral and high local estrogen levels, high 16α-hydroxylated estrogens^b^	Local juxtainflammatory fat deposition
		Low 2-hydroxylated estrogens^c^	Immune activation (16α-hydroxylated forms)
Vitamin D	Bi-modal role: support of innate immunity and inhibition of adaptive immunity [149]	Hypovitaminosis D is common	Osteoporosis, cachexia
			Immune activation toward Th1 and Th17
Osteocalcin	Not known	Little and ambiguous results	Unclear
Vagus nerve	Immunosuppressive in acute inflammation (TNF)	Low activity	Loss of appetite, gastrointestinal disturbances, immune activation
**Energy expenditure hormones**			
Cortisol	Immunosuppressive	Normal to slightly increased in GC-free patients, low levels in GC-pretreated patients	Cachexia, osteoporosis
			Volume overload
			Not much influence on immune system
Sympathetic nervous system (noradrenaline/adrenaline)	β-Adrenergic: suppressive for innate immunity and T helper type 1 lymphocytes, support of B lymphocytes	High activity	Cachexia, osteoporosis
	α-Adrenergic: support of inflammation		Hypertension, volume overload
			Immune activation due to nerve fiber loss^d^
Growth hormone	Immunostimulatory	Little and ambiguous results	Cachexia, osteoporosis
Thyroid hormones (T3)	Directly immunostimulatory	Low T3 levels, diminished activity of the hypothalamic-pituitary-thyroid gland axis but possibly normal T3 levels in muscle	Cachexia
	Indirectly via provision of inorganic iodide		Immune activation in granulocytes
RAAS (angiotensin II)	Directly immunostimulatory	Elevated activity	Volume overload, hypertension
			Cachexia, insulin resistance, osteoporosis
			Immune activation

^a^This will not lead to immunosuppression due to loss of sympathetic nerve fibers in inflamed tissue and secondary lymphoid organs. ^b^These are proproliferative mitogenic estrogens. ^c^These are anti-mitogenic estrogens. ^d^Sympathetic nerve fiber loss was described locally in inflamed tissue and in secondary lymphoid organs. GC, glucocorticoid; IGF-1, insulin-like growth factor-1; RAAS, renin-angiotensin-aldosterone system; T3, tri-iodothyronine; Th, T helper lymphocyte; TNF, tumor necrosis factor.

Although this theory can explain many complications in CIDs, no treatment schemes exist to treat these individual abnormalities in CIDs. The next decade should address treatment rules to overcome these complications because they determine advanced mortality in our patients with CIDs.

## Abbreviations

ACE: Angiotensin-converting enzyme; AT: Angiotensin type; CID: Chronic inflammatory disease; ER: Estrogen receptor; HPA: Hypothalamic-pituitary-adrenal; IFN: Interferon; IGF-1: Insulin-like growth factor-1; IL: Interleukin; RA: Rheumatoid arthritis; RAAS: Renin-angiotensin-aldosterone system; SLE: Systemic lupus erythematosus; SNS: Sympathetic nervous system; T3: Tri-iodothyronine; T4: Thyroxine; TNF: Tumor necrosis factor; TSH: Thyroid gland-stimulating hormone.

## Competing interests

The author declares that he has no competing interests.
